# Validation of bone mineral density measurement using quantitative CBCT image based on deep learning

**DOI:** 10.1038/s41598-023-38943-8

**Published:** 2023-07-24

**Authors:** Chan-Soo Park, Se-Ryong Kang, Jo-Eun Kim, Kyung-Hoe Huh, Sam-Sun Lee, Min-Suk Heo, Jeong-Joon Han, Won-Jin Yi

**Affiliations:** 1 Department of Oral and Maxillofacial Radiology, School of Dentistry, Seoul National University, Seoul, South Korea; 2 Department of Biomedical Radiation Sciences, Graduate School of Convergence Science and Technology, Seoul National University, Seoul, South Korea; 3 Department of Oral and Maxillofacial Radiology and Dental Research Institute, School of Dentistry, Seoul National University, Seoul, South Korea; 4 Department of Oral and Maxillofacial Surgery, School of Dentistry, Seoul National University, Seoul, South Korea

**Keywords:** Computed tomography, Cone-beam computed tomography, Bone imaging

## Abstract

The bone mineral density (BMD) measurement is a direct method of estimating human bone mass for diagnosing osteoporosis, and performed to objectively evaluate bone quality before implant surgery in dental clinics. The objective of this study was to validate the accuracy and reliability of BMD measurements made using quantitative cone-beam CT (CBCT) image based on deep learning by applying the method to clinical data from actual patients. Datasets containing 7500 pairs of CT and CBCT axial slice images from 30 patients were used to train a previously developed deep-learning model (QCBCT-NET). We selected 36 volumes of interest in the CBCT images for each patient in the bone regions of potential implants sites on the maxilla and mandible. We compared the BMDs shown in the quantitative CBCT (QCBCT) images with those in the conventional CBCT (CAL_CBCT) images at the various bone sites of interest across the entire field of view (FOV) using the performance metrics of the MAE, RMSE, MAPE (mean absolute percentage error), R^2^ (coefficient of determination), and SEE (standard error of estimation). Compared with the ground truth (QCT) images, the accuracy of the BMD measurements from the QCBCT images showed an RMSE of 83.41 mg/cm^3^, MAE of 67.94 mg/cm^3^, and MAPE of 8.32% across all the bone sites of interest, whereas for the CAL_CBCT images, those values were 491.15 mg/cm^3^, 460.52 mg/cm^3^, and 54.29%, respectively. The linear regression between the QCBCT and QCT images showed a slope of 1.00 and a R^2^ of 0.85, whereas for the CAL_CBCT images, those values were 0.32 and 0.24, respectively. The overall SEE between the QCBCT images and QCT images was 81.06 mg/cm^3^, whereas the SEE for the CAL_CBCT images was 109.32 mg/cm^3^. The QCBCT images thus showed better accuracy, linearity, and uniformity than the CAL_CBCT images across the entire FOV. The BMD measurements from the quantitative CBCT images showed high accuracy, linearity, and uniformity regardless of the relative geometric positions of the bone in the potential implant site. When applied to actual patient CBCT images, the CBCT-based quantitative BMD measurement based on deep learning demonstrated high accuracy and reliability across the entire FOV.

## Introduction

The number of implant treatment cases in dental clinics is increasing rapidly due to the development of implant technology, the popularization of implant treatment, and an increase in the elderly population^1^. As the number of implant treatment cases increases, many cases of failure and side effects also occur^2^. Based on the 10-year survival rate, the failure rate of implant surgery is about 5%^2,3^, and it rises to 50% when re-implantation is repeated^4^. To reduce the failure rate, accurate pre- and postoperative evaluations of the implant site are essential. The preoperative evaluation of hard tissue at the implant site includes bone quantity and bone quality^5–7^, which are important factors that determine the primary stability after implant placement, which is in turn directly correlated with the implant success rate^8–11^. Bone quality is determined by the bone density and structure, and affects biomechanical properties such as bone strength and the modulus of elasticity^12–16^. Bone quantity can be considered as a three-dimensional evaluation of the amount of bone available in the expected implant placement site and the cortical bone thickness^17^. The postoperative evaluation after implant placement shows bone formation and remodeling between the implant and its surrounding bony interface^8^. To evaluate osseointegration, which can be considered to represent secondary stability, nondestructive test methods such as impact-based techniques or a resonance frequency analysis can be used^18^.

The bone mineral density (BMD) measurement is a direct method of estimating human bone mass for diagnosing osteoporosis, and performed in dental clinics to objectively evaluate bone quality before implant surgery and as part of the peri-implant radiographic evaluation after surgery^19^. Currently dental clinics generally determine the density of the jawbone using cone-beam CT (CBCT)^20–25^. Compared with multi-detector CT (MDCT), CBCT has the advantages of low cost, low radiation dose, and high spatial resolution^26–29^, but it has the disadvantages of high noise, low contrast, and low reliability for bone mineral density (BMD) measurement^30–33^. Compared with MDCT, CBCT also causes physical non-idealities and severe scatter contamination because of differences in the equipment size, geometry of the x-ray source beam, and radiation dose^34,35^. Cupping artifacts and the occurrence of streaks and dark bands damage the image quality and create artifacts, making it difficult to trust the gray values (GVs) and anatomical information obtained from CBCT^36,37^. The GV of voxels in CBCT images can vary due to imaging environmental factors and the inherent limitations of CBCT, such as the geometry of the beam and low dose of radiation^38–43^. In particular, the geometry of the cone beam can cause GV variability between central and peripheral positions and between axial slices within the field of view (FOV), along with asymmetrical endo/exo-mass effects^5,44–46^. As a result, CBCT images are inadequate for quantitatively measuring bone density because it is difficult to calculate accurate Hounsfield units (HU) with GVs obtained by CBCT and to directly compare the bone density of different sites.

Various researchers have tried to solve the problem of inaccurate HU measurement in CBCT images^47–55^. Mah et al. performed two studies to investigate the relationship between GVs and HU in CBCT^56,57^. They established a satisfactory linear relationship between the GVs and the attenuation coefficients, which made it possible to calculate the HU^56,57^. The coefficient of determination (R^2^) for their system was 0.998, and they achieved good results, with a difference of 1–3% between the calculated HU and the actual HU^57^. Cao et al. proposed a model-based framework for artifact correction and image reconstruction to enable the use of CBCT images in quantitative assessments of BMD^58^. Their framework showed good accuracy, with an error within 20 mg/mL in all ranges of concentrations and configurations, and they achieved CVs 20–25% lower than conventional methods, enabling accurate and reproducible BMD measurements^58^. Those studies were performed using phantoms that contained different materials of known composition and density. Clinically, the quantitative use of GVs from CBCT images should generally be avoided, despite attempts to improve the non-uniformity of the CBCT voxel value and the non-linearity of the HU value between CBCT and MDCT images^59,60^.

With the rapid development of artificial intelligence technology, deep learning methodologies have been widely used for image processing in the medical field^61^. A deep learning model developed with a high-quality dataset can be mounted onto actual medical equipment to increase the performance of that equipment, reduce the number of simple repetitive actions required of doctors, and help make clinically useful judgments^62,63^. A generative adversarial network (GAN) model can be used to improve the quality of radiation images, convert the image modality, or generate pathological images^64–69^. A GAN consists of two networks, a generator that generates data and a discriminator that distinguishes fake data, that compete with each other to generate more plausible data^70,71^. U-Net is a network widely applied to segmentation, image quality improvement, or image synthesis^72–75^. It is a U-shaped network that combines an encoder that compresses and down-samples an image with a decoder that up-samples abstract information to make an image of its original size^76^. The U-Net based approach could efficiently synthesize artifact-suppressed images from CBCT images containing global scattering and local artifacts^72,73^.

Previously, we used human skull phantoms and a deep-learning model (QCBCT-NET) to quantitatively measure BMD from CBCT images by enhancing the linearity and uniformity of the bone images^77^. QCBCT-NET substantially enhanced the anatomical and quantitative accuracy of the bone intensities and demonstrated more accuracy than the Cycle-GAN and U-Net models when quantitatively measuring BMD in CBCT images^77^. However, because the previous study used a dataset from human skull phantoms instead of actual patients, it was necessary to evaluate the accuracy and reliability of QCBCT-NET by making BMD measurements from actual patient CBCT images.

Our main hypothesis in this study is that BMD measurements for actual patients made using QCBCT-NET, which is based on deep learning, should show better accuracy and reliability than conventional CBCT image processing across the entire FOV. Therefore, our objective in this study is to validate the accuracy and reliability of BMD measurements made using quantitative CBCT image based on deep learning by applying the method to clinical data from actual patients.

## Materials and methods

### Data acquisition and preparation

This study was approved by the Institutional Review Board of Seoul National University Dental Hospital (No. CRI21010). The object and method of this study was explained, and informed consent was obtained from all subjects. All aspects of the study workflow were conducted in compliance with the relevant guidelines and regulations (Declaration of Helsinki). All MDCT and CBCT images were taken from July 2021 to June 2022 and anonymized to protect personal information. A total of 30 patients (18 males and 12 females; age range, 21–80 years) who needed maxilla and mandible MDCT scans for dental treatment were recruited for this study. The images of the patients were obtained with MDCT (Somatom Definition Edge, Siemens AG, Erlangen, Germany) and CBCT (CS 9300, Carestream Health, Inc., Rochester, US) systems. We acquired the MDCT images at120 kVp and 120 mA with voxel sizes of 0.37 × 0.37 × 0.6 mm^3^, dimensions of 512 × 512 pixels, and 12 bit depth, and we obtained the CBCT images at 80 kVp and 8 mA with voxel sizes of 0.25 × 0.25 × 0.25 mm^3^, dimensions of 669 × 669 pixels, and 16 bit depth.

**Figure 1:**
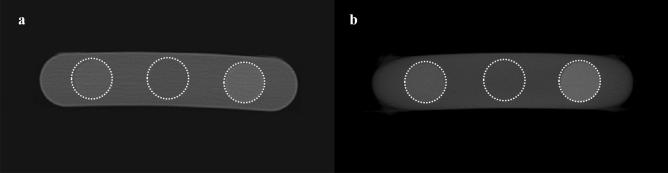
MDCT (**a**) and CBCT (**b**) images of BMD calibration phantom with calcium hydroxyapatite inserts of three densities (0 (center circle), 100 (left circle), and 200 (right circle) mg/cm^3^).

In addition, we also obtained MDCT and CBCT images of a BMD calibration phantom (QRM-BDC Phantom 200 mm length, QRM GmbH, Moehrendorf, Germany) with calcium hydroxyapatite inserts of three densities (0 (water), 100, and 200 mg/cm^3^) under the same conditions (Fig. 1). The MDCT images of the patients were converted into quantitative CT (QCT) images by linearly calibrating the HU in the patient data with the HU in the MDCT images of the BMD calibration phantom. The CBCT images of the patients were also converted into calibrated CBCT (CAL_CBCT) images using the corresponding images of the BMD calibration phantom for comparison. The CAL_CBCT images were afterwards used for comparisons with the results of deep learning (described below)^77^. The MDCT and corresponding CBCT images were matched by paired-point registration using software (3D Slicer, MIT, Massachusetts, US) with manually localized landmarks for deep learning^77^. We prepared 4500 pairs of axial slice QCT and CBCT images from 18 patients for the training and validation datasets, and then we independently prepared another 3000 pairs of QCT and CBCT images from 12 patients for the test dataset.

### QCBCT-NET for quantitative CBCT images

**Figure 2:**
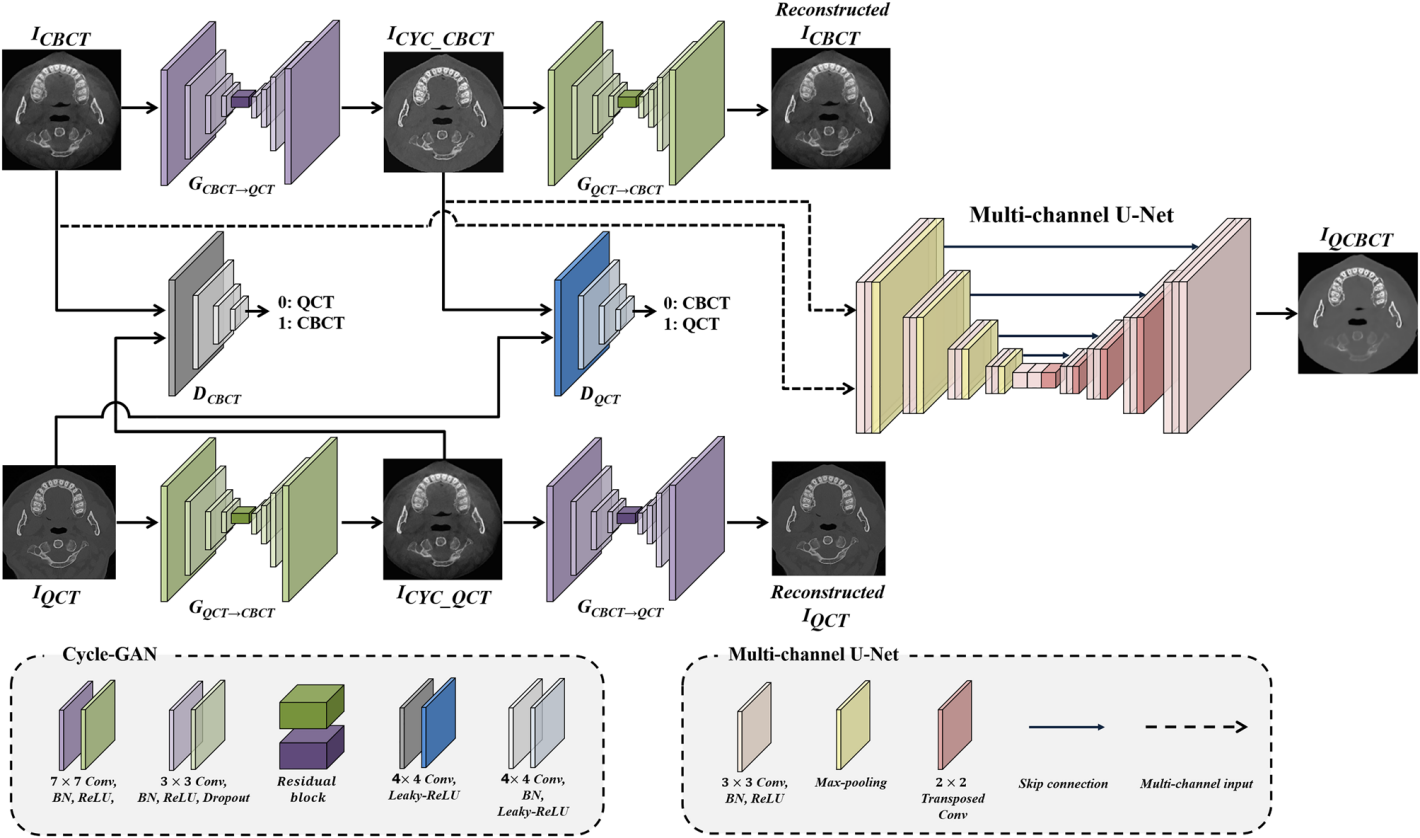
The QCBCT-NET architecture combining Cycle-GAN and the multi-channel U-net^77^. The Cycle-GAN consisted of two generators of *G*_*CBCT⟶QCT*_, and *G*_*QCT⟶CBCT*_, and two discriminators of ${\text{D}}_{\text{CBCT}}$, and ${\text{D}}_{\text{QCT}}$. The multi-channel U-Net had two-channel inputs of CBCT and corresponding CYC_CBCT images, consisting of 3 × 3 convolution layers with batch normalization and ReLU activation, and had skip connections at each layer level. Max-pooling was used for down-sampling and transposed convolution was used for up-sampling. Consequently, the QCBCT-NET generated QCBCT images from CBCT images to quantitatively measure BMD in CBCTs.

In a previous study, we developed a deep-learning model (QCBCT-NET) composed of Cycle-GAN with residual blocks and multi-channel U-Net to generate QCT-like images from conventional CBCT images (Fig. 2)^77^. The Cycle-GAN consisted of two generators, one to map CBCT images to QCT images (G_CBCT→QCT_) and one to map QCT images to CBCT images (G_QCT→CBCT_), and two discriminators to distinguish between real (D_QCT_) and generated (D_CBCT_) images^66,77^. We used a ResNet architecture with nine residual blocks for the generators and PatchGAN for the discriminators^77^. To generate QCBCT images, we constructed a multi-channel U-Net by combining two channel inputs, the original CBCT image and the corresponding output of the Cycle-GAN^77^. The QCBCT-NET was trained with the Adam optimizer using a mini-batch size of 4, an epoch length of 100, and a learning rate of 0.0002. The learning rate was set to 0.0001, and momentum terms were assigned a value of 0.9 to stabilize the training^77^.

### Comparison of BMD measurements from QCBCT and CAL_CBCT images

**Figure 3:**
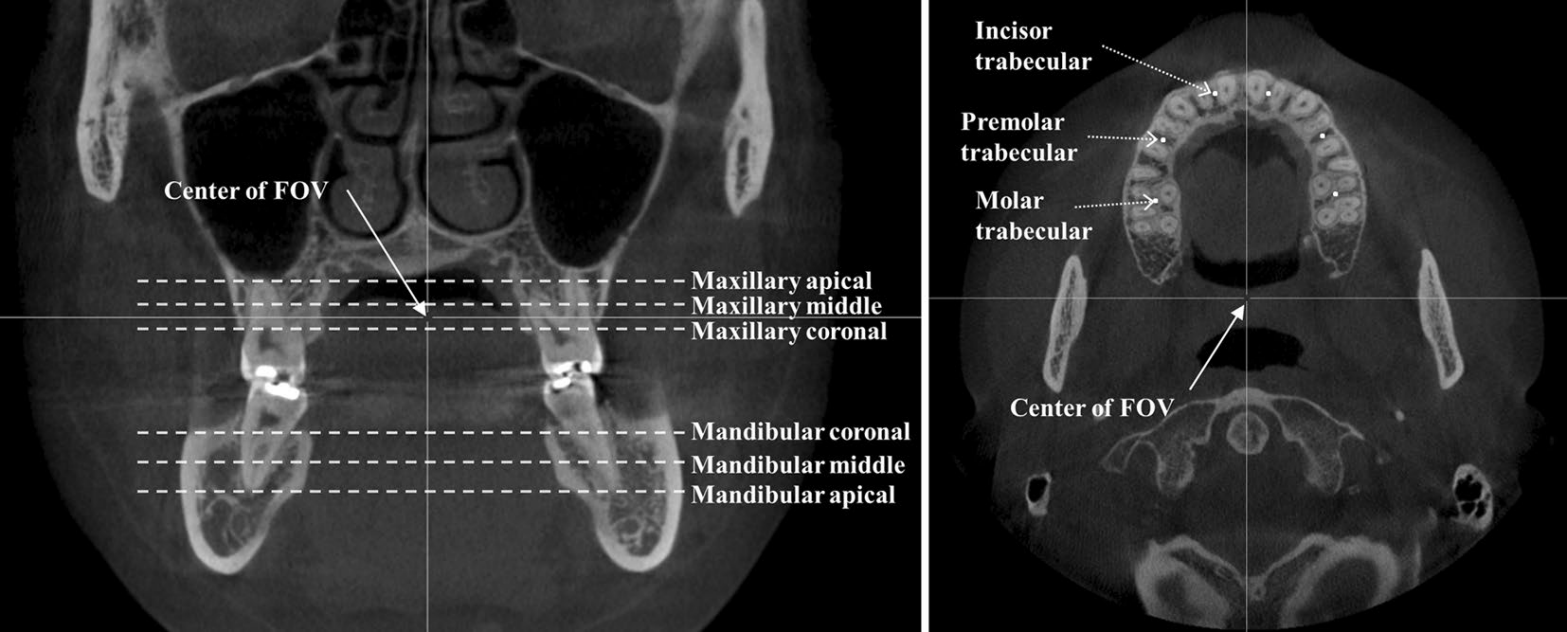
Bone sites of interest at the maxilla and mandible across the entire field of view (FOV) of CBCT. (Left) Axial slices in the coronal, middle, and apical planes along the z-axis in the maxilla and the mandible. (Right) Interdental trabecular bone sites in the left and right incisors, premolars, and molars in same axial plane. The center of the FOV of each CBCT image is indicated by an arrow.

We measured BMDs using QCBCT and CAL_CBCT images at the bone regions of potential implant sites on the maxilla and mandible in the same axial plane and between axial slices across the entire FOV of the CBCT images to simulate a preoperative BMD evaluation. We selected 36 cubic volumes of interest (VOIs) of 5 × 5 × 5 voxels on the maxilla and mandible for each patient. The center points of the VOIs were localized at the trabecular bones of the six interdental bones of the left and right incisors, premolars, and molars in six slices from the coronal, middle, and apical regions of the tooth on both the maxilla and mandible (Fig. 3). The mean BMD of the VOI voxels was used as the representative BMD at the bone region of the potential implant site.

To analyze the accuracy of the BMD measurements made from QCBCT and CAL_CBCT images at various bone sites of interest in the axial plane and between axial slices across the entire FOV, the VOIs at the different bone sites were categorized into five groups (Fig. 3). With regard to the accuracy of the measurements at bone sites between axial slices, the VOIs were categorized in three groups as follows: group A, the maxillary bone and the mandibular bone; group B, the maxillary apical bone, the maxillary middle bone, and the maxillary coronal bone; and group C, the mandibular coronal bone, the mandibular middle bone, and the mandibular apical bone. With regard to the accuracy of the measurements at bone sites in the same axial plane, the VOIs were categorized in two groups as follows: group D, the maxillary incisor bone, the maxillary premolar bone, and the maxillary molar bone; and group E, the mandibular incisor bone, the mandibular premolar bone, and the mandibular molar bone (Fig. 3).

To compare accuracy of BMD measurements between QCBCT and CAL_CBCT images at various bone sites of interest across the entire FOV, we calculated the mean absolute error (MAE), root mean square error (RMSE), mean absolute percentage error (MAPE), correlation coefficient (r), coefficient of determination (R^2^), and standard error of estimation (SEE) between the BMD values from the experimental and QCT (ground truth) images. Paired t-tests were performed on the MAE and MAPE values to determine whether the QCBCT and CAL_CBCT results differed with statistical significance. To evaluate the linearity of the BMD measurements from QCBCT and CAL_CBCT images at various bone sites of interest, a linear regression analysis was performed between the experimental and QCT images, and a Bland–Altman analysis was also performed to compare the agreement between the experimental and QCT results^78^.

To compare accuracy of BMD measurements between different bone sites of interest in the axial plane and between axial slices across the entire FOV, one-way ANOVA tests were performed among MAPEs at the different bone sites for both QCBCT and CAL_CBCT images. When statistical significance was shown in the ANOVA test, the statistically significant differences and relations were identified using a post-hoc analysis (Tukey's HSD test). All tests were performed with a significance level of 0.01 (SPSS v26, SPSS Inc., Chicago, IL, USA).

## Results

**Figure 4:**
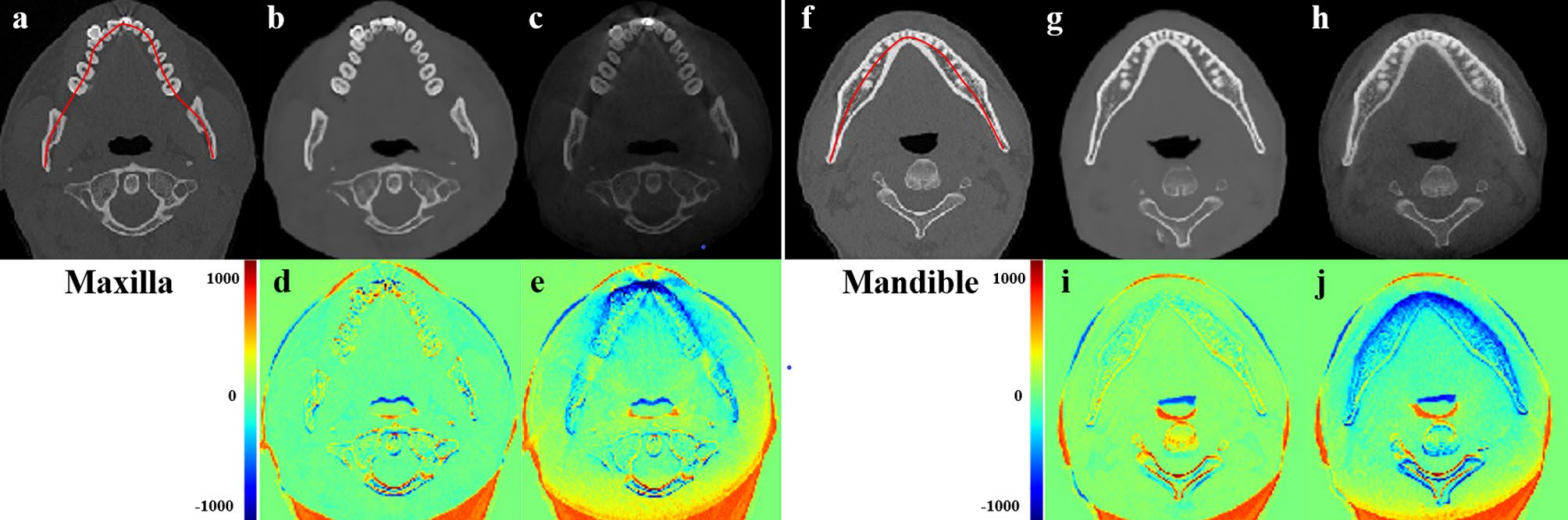
Axial slices of BMD images from the original QCT (**a**,**f**), QCBCT (**b**,**g**), and CAL_CBCT (**c**,**h**) images and their subtractions from the original QCT images at the maxilla (**d**,**e**) and mandible (**i**,**j**). The red curves shown in the QCT images are the dental arch used to obtain the BMD profiles.

**Figure 5:**
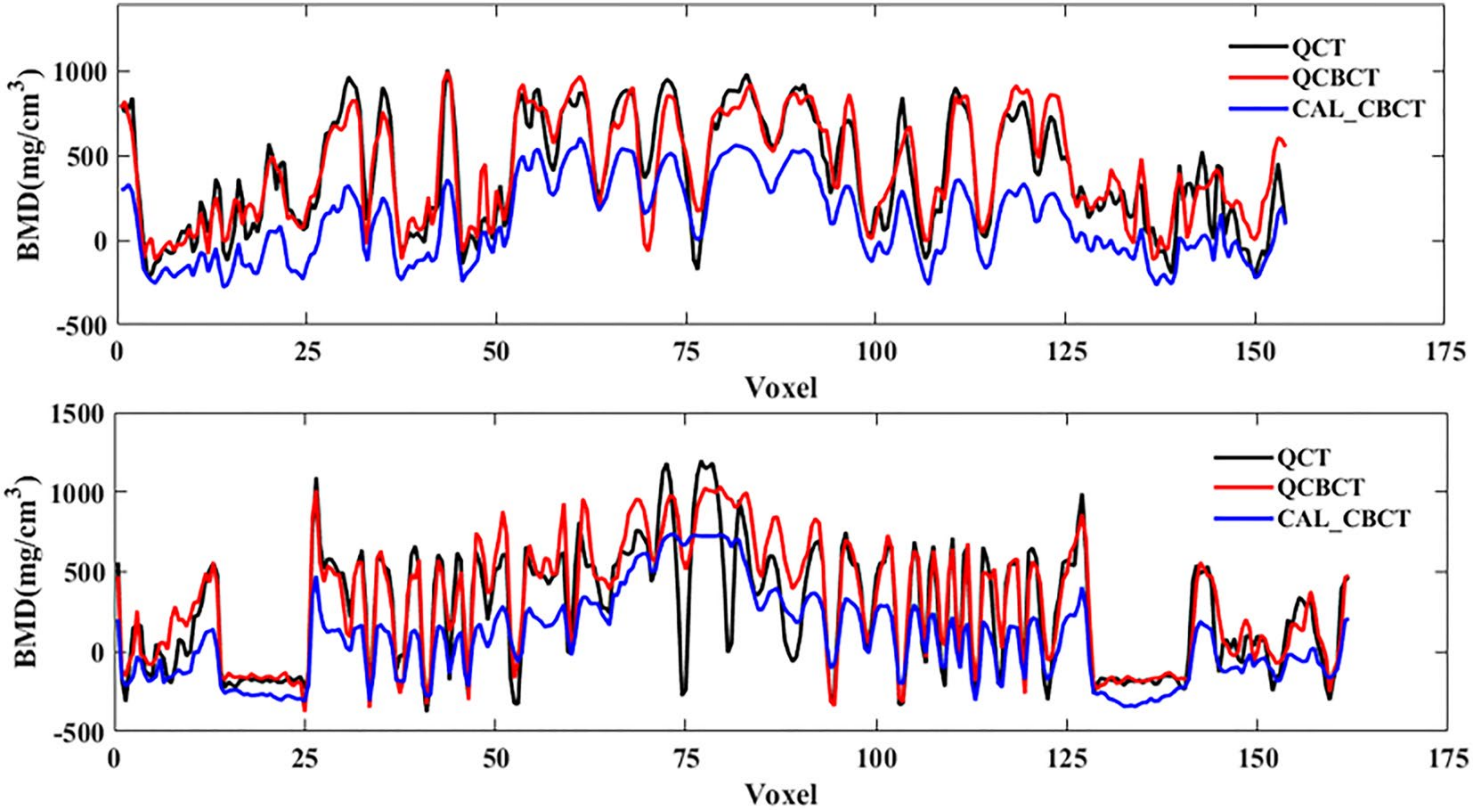
BMD profiles along the dental arch at the maxilla (top) and the mandible (bottom) in the QCT, QCBCT, and CAL_CBCT images shown in Fig. 4. The Pearson correlation coefficients between the QCBCT and CAL_CBCT images and the original QCT images were 0.91 and 0.67, respectively, for the profile at the maxilla and, 0.89 and 0.62, respectively, for the profile at the mandible.

Figure 4 shows axial-slice QCT, QCBCT, and CAL_CBCT images used to calculate BMD at the maxilla and mandible. Compared with the original QCT images, the QCBCT image quality in both regions demonstrated a substantial improvement over that in the CAL_CBCT images in terms of BMD determination. Compared with the CAL_CBCT images, the QCBCT images showed considerably decreased disparities around the teeth and areas of higher BMD. Because complete matching between the CT and CBCT images was not possible because of head positional differences in the patients between the CT and CBCT scans, large differences were observed in the airways, spine, and soft tissues in both the QCBCT and CAL_CBCT images (Fig. 4). Figure 5 shows the BMD profiles that were acquired along the dental arch at the maxilla and mandible in the QCT and experimental images shown in Fig. 4. Compared with that from the CAL_CBCT images, the BMD profile from the QCBCT images more closely resembles and better correlates with that in the original QCT images. Therefore, the QCBCT images show more similarity to the QCT images than the CAL_CBCT images at both the maxilla and mandible.

**Figure 6:**
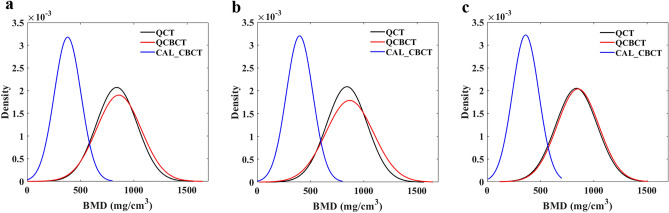
The BMD distributions at both the maxilla and mandible (**a**), the maxilla (**b**), and the mandible (**c**) in the QCT, QCBCT, and CAL_CBCT images.

The results in Table 1 show the accuracy (MAE, RMSE, and MAPE) of the BMD measurements made using the QCBCT and CAL_CBCT images, as compared with the QCT images at various bone sites of interest. The BMD values made using QCBCT measurements showed significantly lower MAE and MAPE values than those made using CAL_CBCT images for all bone sites across the entire FOV (p < 0.01). The mean RMSE of the QCBCT images across all sites was 83.41 mg/cm^3^, whereas that of the CAL_CBCT images was 491.15 mg/cm^3^. The MAE of the QCBCT images showed an overall mean of 67.94 mg/cm^3^, ranging from 63.66 at the mandible to 72.21 at the maxilla, whereas the MAE for the CAL_CBCT images had an overall mean of 460.52 mg/cm^3^, ranging from 444.32 at the maxilla to 476.73 at the mandible. The MAPE of the QCBCT images showed an overall mean of 8.32%, ranging from 7.89 at the mandible to 8.76 at the maxilla, whereas the MAPE of the CAL_CBCT images had an overall mean of 54.29%, ranging from 52.27 at the maxilla to 56.31 at the mandible. Figure 6 shows that the BMD distribution from the QCBCT images was closer to that in the original QCT images than that in the CAL_CBCT images for both the maxilla and mandible. Therefore, the BMD measurements made using the QCBCT images were more accurate than those made using the CAL_CBCT images, regardless of the bone site of interest or anatomical structures of the maxilla and mandible across the entire FOV.

**Table 1:** Accuracy of QCBCT and CAL_CBCT images compared with QCT images for measuring BMD at various bone sites of interest.

Group	Bone site	QCT	QCBCT				CAL_CBCT			
		Mean ± SD (mg/cm^3^)	Mean ± SD (mg/cm^3^)	RMSE (mg/cm^3^)	MAE (mg/cm^3^)	MAPE (%)	Mean ± SD (mg/cm^3^)	RMSE (mg/cm^3^)	MAE (mg/cm^3^)	MAPE (%)
	All	840.41 ± 192.69	860.06 ± 209.88	83.41	67.94 ± 48.46*	8.32 ± 5.99*	379.89 ± 125.49	491.15	460.52 ± 170.92	54.29 ± 13.45
A	Maxilla	842.72 ± 191.08	867.99 ± 223.18	90.43	72.21 ± 54.56*	8.76 ± 6.50*	398.40 ± 124.64	473.36	444.32 ± 163.63	52.27 ± 13.22
	Mandible	838.11 ± 194.70	852.13 ± 195.88	75.75	63.66 ± 41.15*	7.89 ± 5.41*	361.38 ± 123.88	508.32	476.73 ± 176.82	56.31 ± 13.41
B	Mx-Apical	762.13 ± 143.77	768.34 ± 159.92	82.34	66.06 ± 49.50*	8.90 ± 6.72*	328.68 ± 104.55	453.30	433.45 ± 133.62	56.61 ± 12.84
	Mx-Middle	807.34 ± 186.40	825.15 ± 216.57	79.45	62.46 ± 49.45*	7.75 ± 5.47*	388.77 ± 111.20	445.52	418.57 ± 153.68	51.35 ± 11.79
	Mx-Coronal	958.68 ± 183.46	1010.49 ± 214.07	106.96	88.12 ± 61.06*	9.61 ± 7.14*	477.75 ± 111.08	517.91	480.93 ± 193.52	48.87 ± 13.93
C	Mn-Coronal	910.29 ± 201.41	908.81 ± 208.98	66.95	58.12 ± 33.47*	6.63 ± 3.87*	423.46 ± 111.53	522.49	486.83 ± 191.05	52.34 ± 12.76
	Mn-Middle	820.87 ± 180.51	839.34 ± 178.25	77.83	65.51 ± 42.31*	8.15 ± 5.21*	373.39 ± 117.95	474.48	447.49 ± 158.88	54.02 ± 12.47
	Mn-Apical	783.16 ± 181.63	808.23 ± 188.05	81.07	67.36 ± 46.56*	8.90 ± 6.63*	287.29 ± 102.46	526.35	495.87 ± 177.74	62.58 ± 12.85
D	Mx-Incisor	857.72 ± 217.62	863.49 ± 242.40	79.41	65.25 ± 45.57*	7.62 ± 4.79*	368.75 ± 139.90	518.49	488.97 ± 173.67	57.04 ± 13.14
	Mx-Premolar	790.93 ± 173.10	835.05 ± 207.82	95.07	76.44 ± 56.92*	10.11 ± 7.49*	368.43 ± 103.36	447.65	422.49 ± 149.00	52.83 ± 12.46
	Mx-Molar	879.50 ± 170.07	905.44 ± 215.27	95.87	74.95 ± 60.19*	8.54 ± 6.76*	458.01 ± 106.70	450.53	421.49 ± 160.25	46.95 ± 12.22
E	Mn-Incisor	969.26 ± 216.89	965.32 ± 223.84	70.34	62.80 ± 31.90*	6.79 ± 3.74*	371.70 ± 159.45	630.50	597.55 ± 202.57	61.56 ± 15.36
	Mn-Premolar	786.32 ± 137.42	807.98 ± 149.50	84.17	67.22 ± 51.00*	8.62 ± 6.86*	339.58 ± 100.94	463.91	446.73 ± 125.95	56.70 ± 11.61
	Mn-Molar	758.75 ± 149.38	783.08 ± 154.69	71.99	60.96 ± 38.56*	8.27 ± 5.08*	372.84 ± 100.68	403.02	385.91 ± 117.02	50.67 ± 10.64

RMSE, root mean square error; MAE, mean absolute error; MAPE, mean absolute percentage error; Mx, maxilla; Mn, mandible. * Significant differences (p < 0.01) in MAE between QCBCT and CAL_CBCT images, and MAPE between QCBCT and CAL_CBCT images.

**Figure 7:**
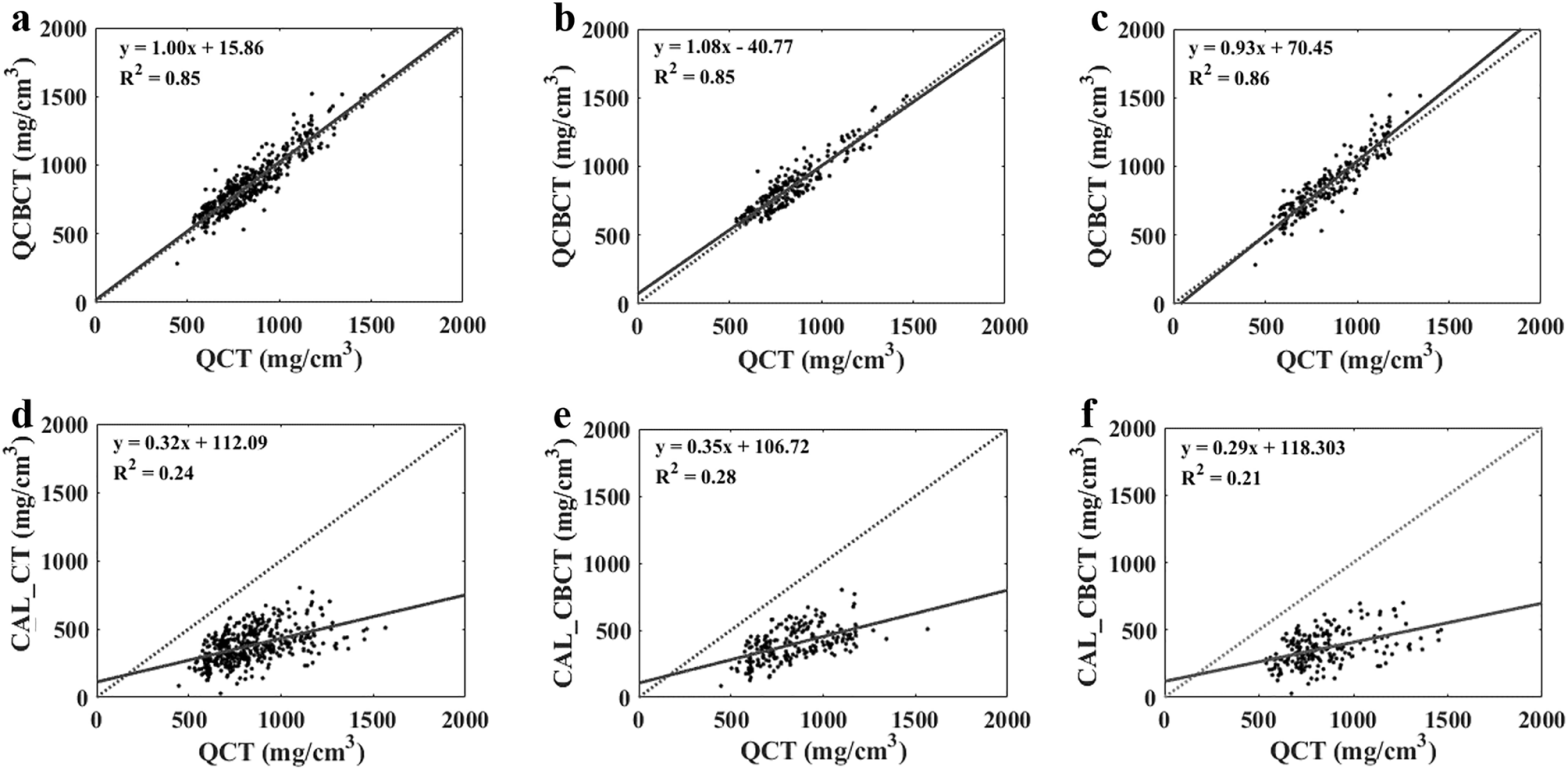
Linear regression plots between QCT (ground truth) and QCBCT (**a**–**c**) images and between QCT (ground truth) and CAL_CBCT images (**d**–**f**) at both the maxilla and mandible (**a**,**d**), the maxilla (**b**,**e**), and the mandible (**c**,**f**).

Table 2 presents the results (r, R^2^, and SEE) from the linearity analysis comparing the BMD values from the QCBCT and CAL_CBCT images with those from the QCT (ground truth) images at various bone sites of interest. The QCBCT images showed closer linear relationships with the QCT results than the CAL_CBCT images at various bone sites across the entire FOV. The linear regression between the QCBCT and QCT images showed an overall slope of 1.00, ranging from 0.93 at the mandible to 1.08 at the maxilla, whereas the regression between the CAL_CBCT and QCT images had an overall slope of 0.32, ranging from 0.29 at the mandible to 0.35 at the maxilla. The overall coefficient of determination for the BMD values from the QCBCT images compared with the QCT images was 0.85, whereas that for the CAL_CBCT images was 0.24. The overall correlation coefficient for the BMD values from the QCBCT images compared those from the QCT images was 0.92, whereas that for the CAL_CBCT images was 0.49. With the larger slope and better goodness of fit, the linear relationship between the QCBCT and QCT images demonstrated greater contrast and correlation than that between the CAL_CBCT and QCT images at the different bone sites (Fig. 7).

**Table 2:** Linearity of QCBCT and CAL_CBCT images compared with QCT images for measuring BMD at various bone sites of interest.

Group	Bone site	QCT	QCBCT					CAL_CBCT				
		Mean ± SD (mg/cm^3^)	Mean ± SD (mg/cm^3^)	Slope	r	R^2^	SEE (mg/cm^3^)	Mean ± SD (mg/cm^3^)	Slope	r	R^2^	SEE (mg/cm^3^)
	All	840.41 ± 192.69	860.06 ± 209.88	1.00	0.92	0.85	81.06	379.89 ± 125.49	0.32	0.49	0.24	109.32
A	Maxilla	842.72 ± 191.08	867.99 ± 223.18	1.08	0.92	0.85	85.53	398.40 ± 124.64	0.35	0.53	0.28	105.41
	Mandible	838.11 ± 194.70	852.13 ± 195.88	0.93	0.93	0.86	73.28	361.38 ± 123.88	0.29	0.46	0.21	110.00
B	Mx-Apical	762.13 ± 143.77	768.34 ± 159.92	0.95	0.86	0.73	81.84	328.68 ± 104.55	0.33	0.46	0.21	92.33
	Mx-Middle	807.34 ± 186.40	825.15 ± 216.57	1.09	0.94	0.88	75.71	388.77 ± 111.20	0.34	0.57	0.32	90.99
	Mx-Coronal	958.68 ± 183.46	1010.49 ± 214.07	1.05	0.90	0.81	93.15	477.75 ± 111.08	0.13	0.21	0.04	107.86
C	Mn-Coronal	910.29 ± 201.41	908.81 ± 208.98	0.98	0.95	0.90	66.84	423.46 ± 111.53	0.20	0.37	0.14	103.01
	Mn-Middle	820.87 ± 180.51	839.34 ± 178.25	0.90	0.91	0.83	73.39	373.39 ± 117.95	0.33	0.50	0.25	101.49
	Mn-Apical	783.16 ± 181.63	808.23 ± 188.05	0.94	0.91	0.83	77.08	287.29 ± 102.46	0.18	0.32	0.10	96.41
D	Mx-Incisor	857.72 ± 217.62	863.49 ± 242.40	1.05	0.95	0.89	78.36	368.75 ± 139.90	0.39	0.60	0.37	110.74
	Mx-Premolar	790.93 ± 173.10	835.05 ± 207.82	1.10	0.92	0.84	82.40	368.43 ± 103.36	0.31	0.52	0.27	87.95
	Mx-Molar	879.50 ± 170.07	905.44 ± 215.27	1.15	0.91	0.83	88.66	458.01 ± 106.70	0.25	0.40	0.16	96.97
E	Mn-Incisor	969.26 ± 216.89	965.32 ± 223.84	0.98	0.95	0.90	70.09	371.70 ± 159.45	0.33	0.45	0.21	141.04
	Mn-Premolar	786.32 ± 137.42	807.98 ± 149.50	0.91	0.84	0.71	80.48	339.58 ± 100.94	0.35	0.48	0.23	88.14
	Mn-Molar	758.75 ± 149.38	783.08 ± 154.69	0.93	0.90	0.81	67.00	372.84 ± 100.68	0.42	0.62	0.39	78.16

Slope, slope of the linear regression; r, correlation coefficient; R^2^, coefficient of determination; SEE, standard error of estimation; Mx, maxilla; Mn, mandible.

**Figure 8:**
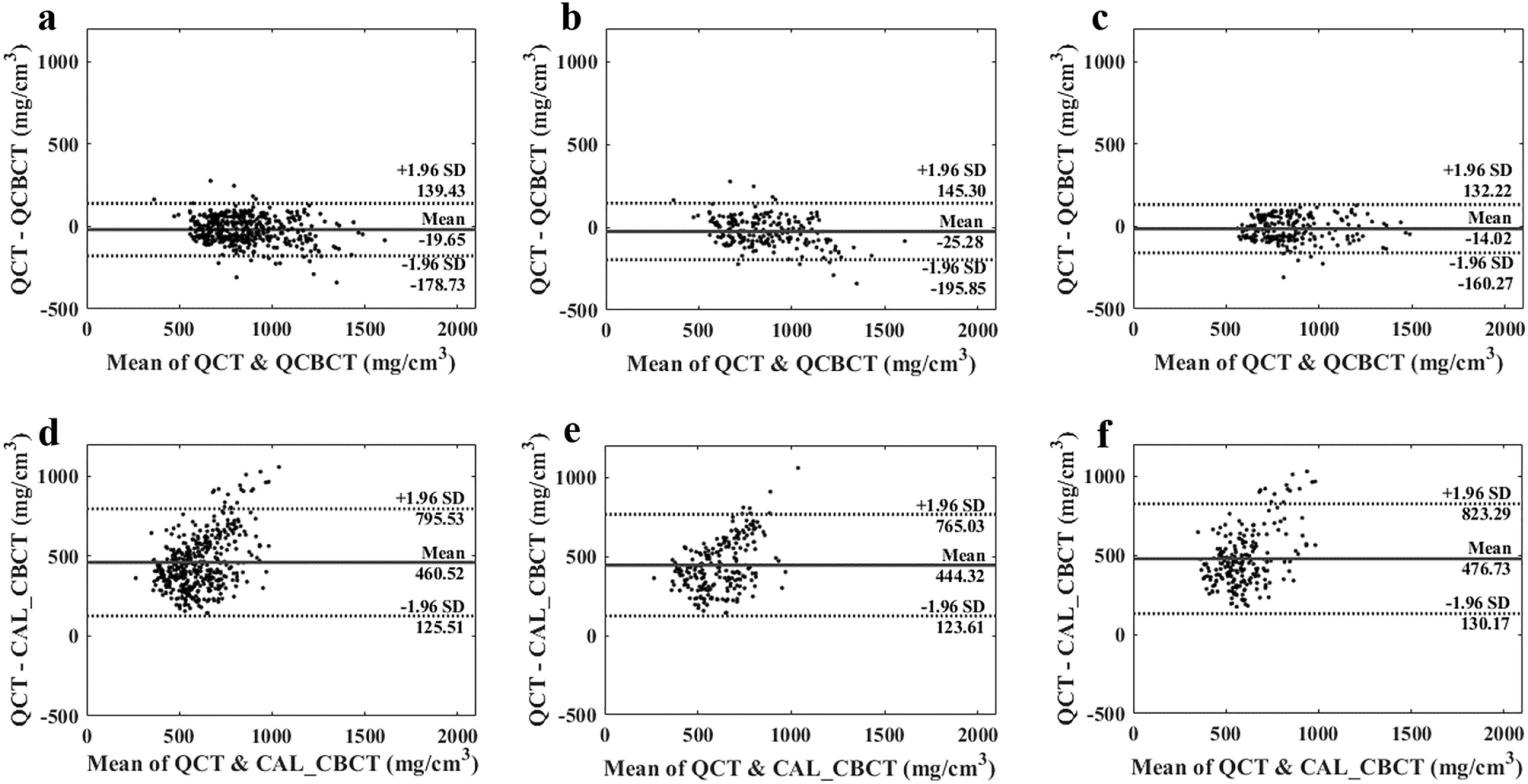
Bland–Altman plots between QCT (ground truth) and QCBCT (**a**–**c**) images and between QCT (ground truth) and CAL_CBCT images (**d**–**f**) at both the maxilla and mandible (**a**,**d**), the maxilla (**b**,**e**), and the mandible (**c**,**f**).

The Bland–Altman plot between the QCBCT and QCT images also demonstrated a more linear relationship and better agreement limits than the plot between the CAL_CBCT and QCT images at the different bone sites (Fig. 8). The mean difference between the QCT and QCBCT images was small, − 19.65 mg/cm^3^, with a relatively uniform distribution around the mean difference value, whereas that between the CAL_CBCT and QCT images was large, 460.52 mg/cm^3^, with an abnormal pattern of an upward slope (Fig. 8). The 95% limits of agreement for the QCBCT images ran from − 178.54 to 139.24, whereas those for the CAL_CBCT were from 125.90 to 795.15 (Fig. 8). Furthermore, the overall SEE for the BMD values from the QCBCT images (compared with the QCT images) was 81.06 mg/cm^3^, whereas that for the CAL_CBCT images was 109.32 mg/cm^3^. Generally, the SEE for the QCBCT images was smaller than that for the CAL_CBCT images at all bone sites of interest, indicating that the QCBCT images enabled BMD measurements with higher uniformity (Table 2). Therefore, the BMD measurements made using the QCBCT images demonstrated more linear relationships, greater contrast, and higher uniformity with the QCT images than those made using the CAL_CBCT images at the various bone sites across the entire FOV.

**Figure 9:**
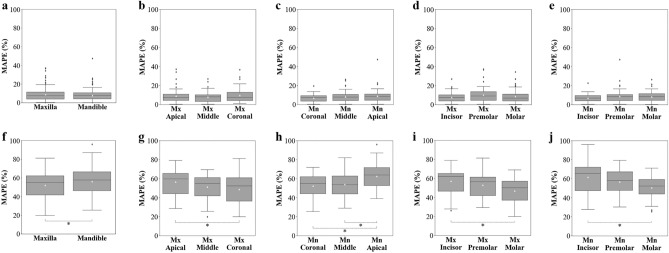
Boxplots for the MAPEs of QCBCT (**a**–**e**) and CAL_CBCT (**f**–**j**) images at various bone regions of interest by group.

The results in Table 3 indicate that the MAPE of the QCBCT images did not differ significantly among the bone sites in all groups (p > 0.01), whereas the MAPE of the CAL_CBCT images did differ significantly among bone sites in all groups (p < 0.01). In the BMD measurements at bone sites between axial slices, the MAPE of the CAL_CBCT images increased in groups A (Maxilla < Mandible), B (Mx-Coronal < Mx-Apical), and C (Mn-Middle, Mn-Coronal < Mn-Apical), as the distance from the center of the FOV increased in the z-axis (p < 0.01) (Table 3). In the BMD measurements at bone sites in the axial plane, the MAPE of the CAL_CBCT images increased in groups D (Mx-Molar < Mx-Incisor) and E (Mn-Molar < Mn-Incisor) as the distance from the center of the FOV increased in the xy-plane (p < 0.01) (Table 3). The center of the FOV in all the CBCT images was between the middle and the coronal site of the maxillary tooth in the z-axis, and in the soft palate region behind the molars in the xy-plane (Fig. 3). On the other hand, the MAPE of the QCBCT images did not differ significantly among bone sites in the axial plane or between axial slices (p > 0.01). Furthermore, the BMD measurements made using the QCBCT images show higher MAPE performances than those made using the CAL_CBCT images, with a smaller dispersion of data and shorter whisker lengths in the boxplots across all bone sites of interest (Fig. 9). Therefore, the BMD measurements made using the QCBCT images demonstrated better reliability than those made using the CAL_CBCT images without regard to bone sites in the axial plane or between axial slices across the entire FOV.

**Table 3:** Results of one-way ANOVA and Tukey’s HSD for MAPEs of QCT and QCBCT images used to measure BMD at various bone sites of interest.

Group	Bone site	QCBCT			CAL_CBCT			
		MAPE (%)	F statistic	p-value	MAPE (%)	F statistic	p-value	Relationship
A	Maxilla	8.76	2.2572	0.1337	52.27*	9.9214	0.0017	Maxilla < Mandible
	Mandible	7.89			56.31			
B	Mx-Apical	8.90	1.5060	0.2241	56.61	6.7957	0.0014	Mx-Coronal < Mx-Apical
	Mx-Middle	7.75			51.35			
	Mx-Coronal	9.61			48.87*			
C	Mn-Coronal	6.63	3.3374	0.0374	52.34*	13.4831	0.0000	Mn-Middle, Mn-Coronal < Mn-Apical
	Mn-Middle	8.15			54.02*			
	Mn-Apical	8.90			62.58			
D	Mx-Incisor	7.62	2.7407	0.0668	57.04	11.6489	0.0000	Mx-Molar < Mx-Incisor
	Mx-Premolar	10.11			52.83			
	Mx-Molar	8.54			46.95*			
E	Mn-Incisor	6.79	2.3530	0.0975	61.56	13.2893	0.0000	Mn-Molar < Mn-Incisor
	Mn-Premolar	8.62			56.70			
	Mn-Molar	8.27			50.67*			

MAPE, mean absolute percentage error; Mx, maxilla; Mn, mandible.

*Significant difference (p < 0.01) among MAPEs at sites of interest within group.

## Discussion

The BMD measurement is a direct method of estimating human bone mass for diagnosing osteoporosis, and performed to objectively evaluate bone quality before implant surgery in dental clinics. The objective of this study was to validate the accuracy and reliability of BMD measurements made using quantitative cone-beam CT (CBCT) image from actual patients based on deep learning. In a previous study, we measured BMD directly and quantitatively from CBCT images using a developed deep-learning model (QCBCT-NET) and human skull phantoms^77^. The QCBCT-NET greatly increased the linearity and uniformity of CBCT images, compared with the Cycle-GAN and U-Net deep learning models, which we demonstrated by showing improvements in quantitative performance^77^. In this study, we evaluated the accuracy and reliability of BMD measurements from QCBCT images created by applying QCBCT-NET to clinical data from actual patients.

We analyzed and compared the accuracy of BMD measurements between CAL_CBCT and QCBCT images from various bone sites of interest across the entire FOV. The QCBCT images showed better accuracy and uniformity and improved linearity and contrast compared with the CAL_CBCT images across the entire FOV. The QCBCT images had an RMSE of 83.41 mg/cm^3^, an MAE of 67.94 mg/cm^3^, and a MAPE of 8.32% for all bone sites of interest across the entire FOV, whereas the CAL_CBCT images had an RMSE of 491.15 mg/cm^3^, an MAE of 460.52 mg/cm^3^, and a MAPE of 54.29%. The linear regression between the QCBCT and QCT images showed a slope of 1.00 and a coefficient of determination of 0.85, whereas those values with the CAL_CBCT images were 0.32, and 0.24, respectively. The correlation coefficient for the BMD values from the QCBCT images, compared with the QCT images, was 0.92, whereas that from the CAL_CBCT image was 0.49. The overall SEE between the QCBCT images and the QCT images was 81.06 mg/cm^3^, whereas that for the CAL_CBCT images was 109.32 mg/cm^3^. Therefore, the BMD measurements made using the QCBCT images had high accuracy and reliability, regardless of the relative geometric positions of the bone across the entire FOV of the CBCT.

The results from the Bland–Altman analysis indicate that compared with the CAL_CBCT images, QCBCT images had a smaller MAE with the QCT images and showed a relatively uniform distribution around the mean difference values in all BMD ranges, indicating better agreement. The CAL_CBCT images showed a significantly upward slope pattern in the Bland–Altman plot, whereas the QCBCT showed a slight downward slope pattern. As the mean BMD values from the QCT and QCBCT images increased, it appears that the size of the error increased proportionately in the plot. Therefore, it would be more beneficial to use the MAPE, a scale-independent accuracy metric, instead of the MAE when comparing the accuracy of BMD measurements between bone sites with different BMD values across the entire FOV.

To evaluate differences in BMD measurement accuracy between various bone sites in the axial plane and between axial slices across the entire FOV of CBCT, we compared the MAPEs at bone sites of interest according to the categorized groups. The MAPEs of the QCBCT images did not differ significantly among bone sites in any group across the entire FOV; however, the CAL_CBCT images showed significant differences in all groups. In the CAL_CBCT images, the accuracy at bone sites of interest in the axial plane and between axial slices decreased as the site of interest became farther from the center of the FOV. Between axial slices, the cone-shaped beam geometry of CBCT means that the incident beam angle is not parallel as the target becomes farther away from the center of the FOV, which causes a decrease in accuracy and an increase in the variability of BMD measurements^42^. Pauwels et al. argued that image quality degradation and artifacts can occur more in the top and bottom parts of the FOV due to the wide cone angle in some CBCT images^40^, which is consistent with our findings for the CAL_CBCT images. In the axial plane, the beam geometry of CBCT causes asymmetry in the X-ray path, leading to position-dependent differences in beam hardening and endo/exo-mass effects and a consequent difference in BMD accuracy between the central and peripheral parts of the FOV^79–81^. Plachtovics et al. showed that the difference between the HU of MDCT and the GV of CBCT increased from the center of the FOV to the outer border within an axial slice, resulting in a decrease in the density measurement accuracy of CBCT, which is consistent with our findings for the CAL_CBCT images^82^.

The significant difference in MAPE values between the bone sites in the CAL_CBCT images, both within the axial plane and between axial slices, suggests the nonlinearity and non-uniformity caused by the cone beam geometry of CBCT. In contrast, no significant differences in MAPE values were found among bone sites across the entire FOV in the QCBCT images, which indicates that the inherent nonlinearity and non-uniformity of CBCT was reduced, which improved the resulting BMD measurements. Therefore, the BMD measurements made using the QCBCT images demonstrated high reliability without regard to the relative geometric positions of the bone sites across the entire FOV. On the other hand, the MAPE of the QCBCT images showed larger values in the premolar and molar bones than the incisor bone at both the maxilla and mandible, but they did not showed significant differences. As a result, the artifacts of metallic or resin restorations more common in the premolars and molars might have the impact of decreasing the reliability on the quality of the QCBCT images in patients.

QCBCT images will bring great progress in measuring bone density and bone mass using CBCT in dental clinical practice and enable the accurate, quantitative evaluation of bone density using only CBCT equipment. We have shown that it can be applied to actual patient clinical data for use in dental implant treatment. It can also help to more accurately estimate the dimension of residual bone and cortical bone thickness during the preoperative evaluation. An accurate preoperative evaluation of bone quality and quantity at the site for implant placement can help to predict the primary implant stability and determine whether implant placement is possible^83,84^. By using this information to make a treatment plan, the failure rate of dental implant placements could be reduced. The secondary stability of an implant is determined by the degree of apposition between the bone and the implant, which is affected by the macro- or micro-architecture of the surrounding bone after implant placement^85^. Therefore, QCBCT images can be helpful in indirectly and easily assessing the secondary stability of implants by accurately measuring the degree of osseointegration at the implant/bone interface after implant surgery. For example, in the case of complications such as a sharp decrease in bone density around the implant in a QCBCT image obtained after surgery, a rapid and precise clinical response will be possible.

By using QCBCT images, we obtained BMD values with improved quantitative accuracy, linearity, and uniformity at the bone regions of a potential implant site across the entire FOV, compared with conventional CBCT images. Overall, the use of QCBCT images can provide more accurate BMD measurements over a larger FOV than conventional CBCT images, making it a valuable tool for assessing bone quality and density in a variety of clinical settings. Further research is needed on the use of QCBCT images to assess bone quality and density for diagnosing and treating various human organs and structures.

This study has some limitations. The first concerns the accuracy of registration. It is difficult to obtain identical images from CBCT and MDCT due to differences in the scanning posture and scanning environment when acquiring the datasets, and fine human errors can occur when setting landmarks in both images during the registration process. When testing the model performance by sampling the sites of interest, an inaccurate part of the registration can lead to image-to-image anatomical inconsistencies, resulting in poor evaluation results. The second limitation is the diversity of the datasets. Lack of diversity in datasets causes bias in deep learning models and increases the risk of overfitting^86^. There is a need to train and evaluate deep learning models with various datasets acquired from multiple institutions using multiple types of equipment^87^. The third limitation is the number of patients. It is necessary to increase the number of the patients requiring various dental treatments to demonstrate the performance of the developed method more definitely and enable its use in various clinical environments. Finally, the artifacts of metallic or resin restorations present in the oral cavity of actual patients^50^ can degrade the quality of QCBCT images, and lead to inaccurate BMD measurements. However, there was a lack of comprehensive analysis for the potential impact of the artifacts on the measurement results in this study. Therefore, it is important to be aware of the potential impact of decreasing the reliability on the quality of the QCBCT images in patients when interpreting the QCBCT images.

## Conclusions

When applied to actual patient CBCT images, quantitative CBCT images for BMD measurement based on deep learning demonstrated high accuracy and reliability at various bone regions around potential implant sites across the entire FOV of CBCT. Therefore, quantitative CBCT–based BMD measurement can be a valuable tool for assessing bone quality and density when diagnosing and treating various human organs and structures by providing high-quality BMD images that can aid in accurate and effective treatment.

## Data Availability

The datasets generated and/or analyzed during the current study are not publicly available due to the restriction by the Institutional Review Board (IRB) of Seoul National University Dental Hospital in order to protect patients’ privacy but are available from the corresponding author on reasonable request. Please contact the corresponding author for any commercial implementation of our research.
